# Soluble *IL6R* represents a miR-34a target: potential implications for the recently identified IL-6R/STAT3/miR-34a feed-back loop

**DOI:** 10.18632/oncotarget.4334

**Published:** 2015-06-02

**Authors:** Huihui Li, Matjaz Rokavec, Heiko Hermeking

**Affiliations:** ^1^ Experimental and Molecular Pathology, Institute of Pathology, Ludwig-Maximilians-Universität München, Munich, Germany; ^2^ German Cancer Consortium (DKTK), Heidelberg, Germany; ^3^ German Cancer Research Center (DKFZ), Heidelberg, Germany

**Keywords:** miR-34a, colorectal cancer, IL-6R, STAT3, inflammation

## Abstract

We previously reported that IL-6R, STAT3 and miR-34a form a positive feedback-loop, which promotes epithelial to mesenchymal transition (EMT), invasion, and metastasis of colorectal cancer (CRC) [1]. In that study only the membrane-bound form of the IL-6R was shown to be repressed by miR-34a. Here, we show that also the mRNA encoding the soluble *IL6R (s-IL-6R*) is directly targeted and repressed by miR-34a. Accordingly, the concentration of s-IL6R protein was decreased in conditioned media of CRC cell lines ectopically expressing miR-34a. The s-IL-6R mediates IL-6 trans-signaling, which also affects cells that do not express the IL-6R. Since IL-6 trans-signaling is involved in numerous inflammatory disease states these findings may be relevant for future therapeutic approaches.

## INTRODUCTION

**Figure 1:**
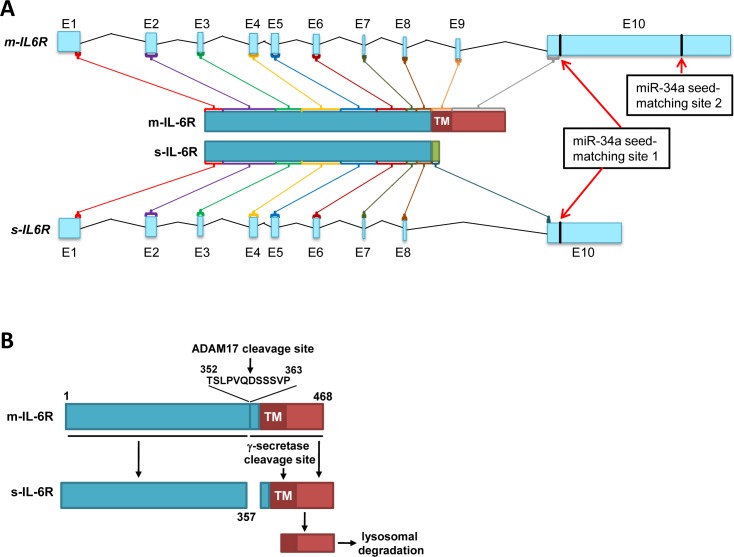
Generation of soluble and membrane IL-6R mRNA and protein isoforms **A.** Generation of IL-6R protein isoforms by alternative splicing. The organization of two alternatively spliced human *IL6R* mRNA products and the resulting proteins is depicted. Protein-coding ORFs are indicated by different colors and are shown as final protein products in the center. The miR-34a seed-matching sequences are indicated with red arrows. The scheme is based on data from *Ensembl* (release 78 - December 2014), Genome assembly: GRCh38 (Homo sapiens). **B.** Generation of soluble IL-6R by cleavage of m-IL-6R protein.

Signaling mediated via the interleukin-6 (IL-6)/interleukin-6 receptor (IL-6R) plays a pivotal role during immune responses and in cancer [2-4]. Upon binding of IL-6, IL-6R associates with gp130, which initiates intracellular signal transduction via the JAK/STAT and the Ras/MAPK/AKT pathways [5]. Besides classical IL-6 signaling, which involves the membrane-bound IL-6R (m-IL-6R), also IL-6 trans-signaling has been described [6]. The later involves the s-IL-6R, which is shedded/released by cells and in complex with IL-6 binds to and activates gp130. Thereby, IL-6 trans-signaling can affect cells that do not express the IL-6R [7]. The human s-IL-6R protein is either generated by skipping exon 9 via alternative splicing and subsequent earlier termination of translation caused by a stop codon in the alternative reading frame in exon 10 (Figure 1A) or by cleavage of the m-IL-6R protein by the α-secretase ADAM17 upstream of the transmembrane (TM) domain (Figure 1B) [8]. The N-terminal IL-6R fragment is shedded, whereas the C-terminal fragment is cleaved by γ-secretase in the transmembrane (TM) domain and removed by lysosomal degradation.

The genes encoding the microRNAs (miRNAs) miR-34a and miR-34b/c are direct p53 target genes and mediate tumor suppressive effects of p53 (reviewed in [9, 10]). miR-34a/b/c have been shown to inhibit cell cycle progression, angiogenesis, stemness, invasion and metastasis by directly down-regulating the expression of key factors promoting these processes. For example, the direct repression of the EMT-TF SNAIL contributes to mesenchymal-epithelial transition induced by p53 [11]. Also Cyclin E, SIRT1, MYC and Wnt/TCF7, Yin Yang 1, L1CAM and c-Kit have been shown to represent direct miR-34 targets, which mediate its tumor suppressive effects [12-18]. Furthermore, miR-34 mimetics are currently being tested for their therapeutic value in a phase 1 trial [19]. In addition, the inactivation of *miR-34a* by CpG methylation may be used for prognostic purposes [20].

Recently, we showed that m-IL-6R is part of a positive feedback loop involving miR-34a and STAT3 [1], which was commented on in [21]. We identified the *IL6R* mRNA as a direct target of miR-34a and showed that *miR-34a* is directly repressed by STAT3. The activation of this loop was required for EMT, invasion, and metastasis and is associated with nodal and distant metastasis in colorectal cancer (CRC) patients.

## RESULTS

**Figure 2:**
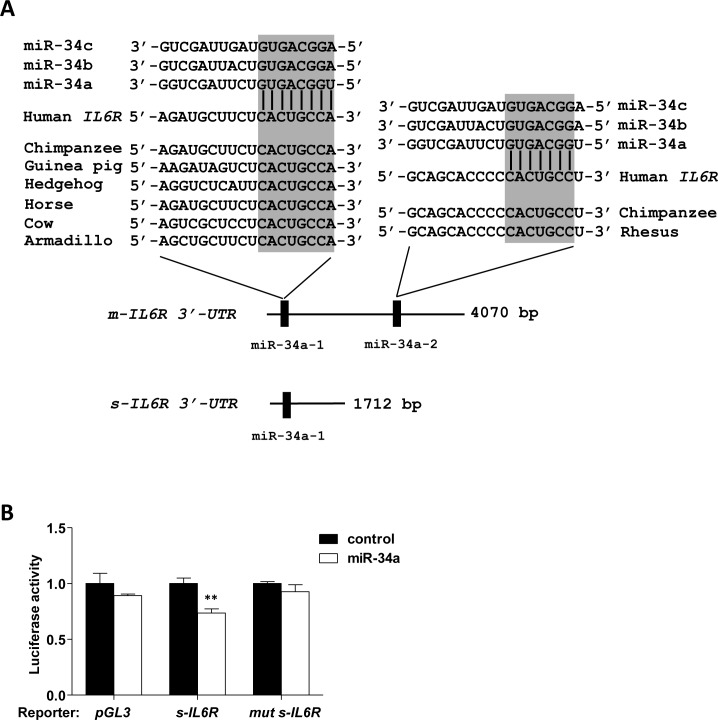
Direct regulation of *s-IL6R* by miR-34a **A.** Schematic representation of the 3′-UTRs of human membrane-bound and soluble *IL6R* mRNA isoforms indicating the miR-34 seed-matching sequences and their phylogenetic conservation. **B.** Dual reporter assay after transfection of H1299 cells with pre-miR-34a oligonucleotides and human s-*IL6R* 3-UTR reporter constructs.

**Figure 3:**
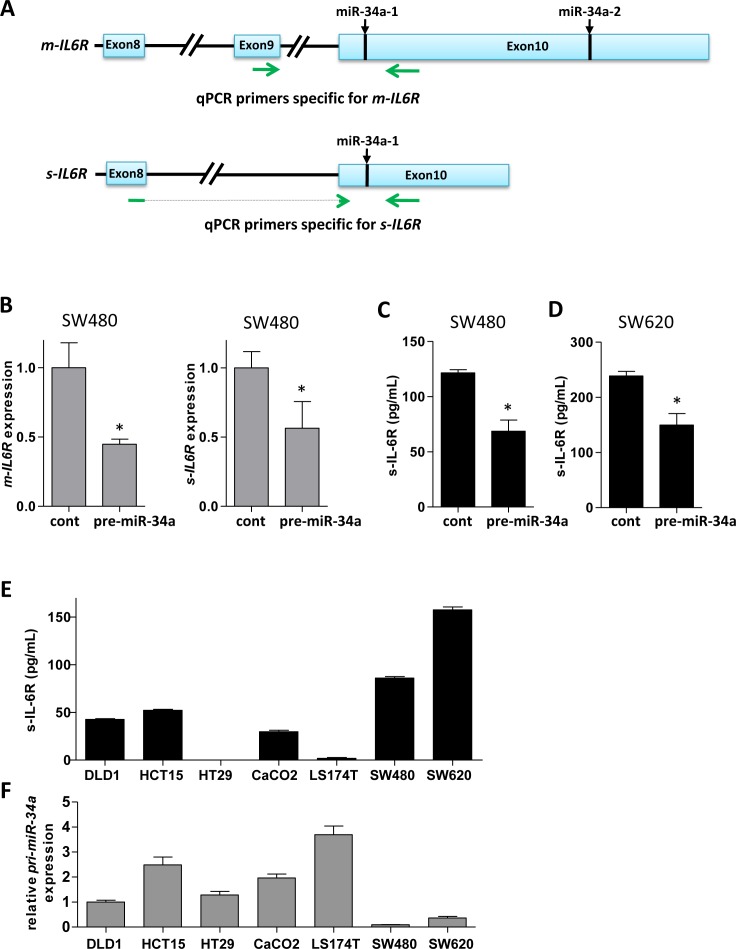
miR-34a down-regulates s-IL-6R expression in CRC cell lines **A.** Location of qPCR primers specific for mRNA isoforms encoding membrane-bound and soluble *IL6R*, respectively. Exon 9 encodes the transmembrane domain, which is absent in the soluble *IL6R* isoform. miR-34a seed-matching sites are indicated. **B.** Expression of m- and s-*IL6R* mRNA in SW480 cells transfected with control or pre-miR-34a oligonucleotides. **C.**, **D.** Expression of s-IL-6R protein in the indicated cell lines transfected with control or pre-miR-34a oligonucleotides. After 72 hours medium was changed and 24 hours later conditioned media was subjected to ELISA. **E.** Expression of s-IL-6R protein in indicated cell lines. Equal numbers of cells was seeded and conditioned media was analyzed by s-IL-6R specific ELISA 48 hours later. **F.** Expression of primary ***miR-34a (pri-miR-34a)*** in the indicated cell lines relative to expression in DLD1 cells.

Here we asked whether the s-IL-6R is a direct target of miR-34a. Based on Ensembl, the s-*IL6R* mRNA isoform has a shorter 3′-UTR sequence than the m-*IL6R* isoform. Yet, it contains a miR-34a seed-matching site, which corresponds to the first site in the m-*IL6R* encoding mRNA (Figure 2A). We found that ectopic miR-34a resulted in repression of a s-*IL6R* 3′-UTR reporter in a dual reporter assay (Figure 2B). This repression was alleviated by mutation of the miR-34a seed-matching site (Figure 2B). To investigate whether also the endogenous transcript isoform that encodes *s-IL-6R* is regulated by miR-34a, we designed qPCR primers that exclusively recognize the *m-IL-6R* or the *s-IL6R* mRNA isoform (Figure 3A). Indeed, ectopic expression of miR-34a resulted in a decrease of the membrane-bound and soluble *IL6R* isoforms in SW480 cells (Figure 3B). Next, we determined the effect of ectopic miR-34a expression on s-IL-6R protein expression by transfecting SW480 and SW620 cells with pre-miR-34a oligonucleotides. s-IL-6R specific ELISA analyses showed that in both cell lines ectopic expression of miR-34a significantly decreased the concentration of s-IL-6R in conditioned media (Figure 3C, 3D). Finally, we showed that the levels of s-IL-6R are elevated in the conditioned media of mesenchymal-like SW480 and SW620 CRC cells that express low levels of miR-34a, whereas epithelial DLD1, HCT15, HT29, CACO2, and LST174 cells express high levels of miR-34a (Figure 3E, 3F). These latter results are in agreement with a previous publication showing that SW620 cells secrete more s-IL-6R than DLD1 and HT29 cells [22].

In addition, it should be mentioned that the NCBI GenBank lists an additional *IL6R* isoform that lacks exon 9, which encodes the transmembrane domain (NM_181359.2). The s-*IL6R* mRNA isoform also lacks exon 9. Thus, the NM_181359.2 mRNA may also encode the *s-IL-6R* protein. However, this isoform contains the full length 3′-UTR and therefore the two miR-34a binding sites as the m-*IL6R* isoform, which we have shown to mediate direct repression of *IL6R* by miR-34a [1].

## DISCUSSION

**Figure 4:**
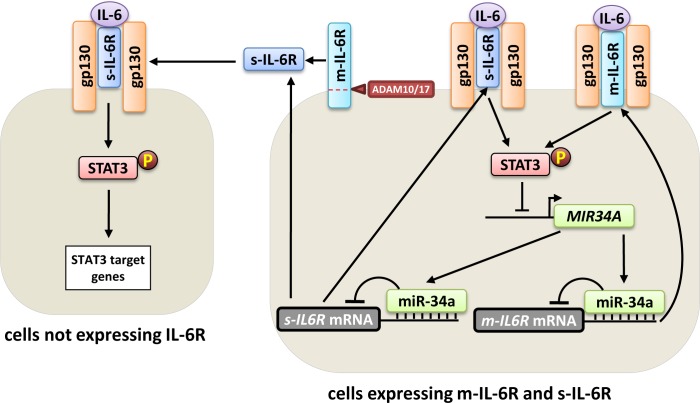
Effect of miR-34a on m-IL-6R and s-IL-6R mediated signaling Right side: In cells that express both, m-IL-6R and s-IL-6R, such as macrophages, miR-34a represses the expression of both IL-6R isoforms. Left side: The regulation of s-IL-6R expression by miR-34a does not only influence cells expressing IL-6R, but may also regulate STAT3 signaling in cells that do not express IL-6R and are responsive only to IL-6 trans-signaling, such as smooth muscle cells, endothelial cells, and keratinocytes.

IL-6 trans-signaling, which is mediated via the s-IL-6R, is critically involved in several inflammatory and autoimmune diseases including inflammatory bowel disease, rheumatoid arthritis, and asthma, as well as colitis-associated cancer [23]. Therefore, understanding the regulation of IL-6 trans-signaling could represent a basis for development of novel drugs that might be useful for treatment of several diseases. Recently, we identified the m-IL-6R as a direct target of miR-34a [1]. Here we extend our previous findings and demonstrate that also the s-IL-6R is a direct target of miR-34a. We show that not only the s-IL-6R that is generated by proteolytic cleavage of m-IL-6R, but also the s-IL-6R that is produced by the *s-IL6R* mRNA isoform are directly targeted and repressed by miR-34a. Taken together, our results show that miR-34a represses the expression of *s-IL6R* mRNA and protein. Therefore, our previous findings about the IL-6R/STAT3/miR-34a loop [1] may be extrapolated to the s-IL-6R isoform and may have implications for IL-6 trans-signaling (Figure 4). MiR-34a mediated repression of s-IL-6R in cells that produce s-IL-6R, such as macrophages may thus regulate IL-6 JAK/STAT signaling also in cells that do not produce m-IL-6R, such as smooth muscle cells, endothelial cells, and keratinocytes [7]. Accordingly, miR-34a suppression, which is common in cancer cells, and consequent enhanced production of s-IL-6R could activate JAK/STAT signaling in stromal cells of the tumor microenvironment. Finally, miR-34a mimetics may be used to repress s-IL-6R expression and consequently IL-6 trans-signaling, to treat inflammatory diseases and cancer.

## MATERIALS AND METHODS

### Cell culture and reagents

DLD-1, HCT-15, HT29, Caco-2, LS174T, SW480, and SW620 CRC cell lines were maintained in McCoy's 5A Medium (Invitrogen) containing 10% FBS. Precursor microRNA oligonucleotides (Ambion pre-miR-34a [PM11030]) were transfected at a final concentration of 25 nM using Lipofectamine 2000 transfection reagent (Invitrogen) according to manufacturer's instructions.

### s-IL-6R-specific ELISA

At indicated time points, conditioned media was collected and subjected to s-IL-6R specific ELISA (R&D, DR600). The analyses were performed according to manufacturer's instructions. In all experiments equal number of cells was seeded.

### RNA isolation and qPCR

Total RNA was isolated using the Total RNA Isolation Kit (Roche) according to the manufacturer's instructions. cDNA was generated from 1 μg total RNA per sample using the Verso cDNA synthesis kit (Thermo Scientific). qPCR was performed by using the LightCycler 480 (Roche) and the Fast SYBR Green Master Mix (Applied Biosystems). mRNA expression was normalized using detection of *GAPDH*. Results are represented as fold induction using the ΔΔCt method [24] with the control set to 1. Oligonucleotides: *GAPDH* (fwd: 5′-GCTCTCTGCTCCTCCTGTTC-3′, rev: 5′-ACGACCAAATCCGTTGACTC-3′), *pri-miR-34a* (fwd: 5′-CGTCACCTCTTAGGCTTGGA-3′, rev: 5′-CATTGGTGTCGTTGTGCTCT-3′), *m-IL6R* (fwd: 5′-CTCCTCTGCATTGCCATTGT-3′, rev: 5′- TGTGGCTCGAGGTATTGTCA-3′), *s-IL6R* (fwd: 5′-CGACAAGCCTCCCAGGTTCA-3′, rev: 5′-CGGTTGTGGCTCGAGGTATT-3′).

### Dual reporter assays

The 3′-UTR of human *s-IL6R* was PCR amplified from oligo-dT-primed cDNA of human diploid fibroblasts with the Verso cDNA kit (Thermo Scientific), inserted into pGL3-control-MCS, and verified by sequencing. Mutations in the miR-34a seed-matching sequence were generated with the QuikChange Mutagenesis Kit according to the manufacturer's instructions (Stratagene). For luciferase assays, H1299 cells were seeded in 12-well format dishes with 3 × 10^4^ cells/well and transfected after 24 hours with 100 ng of the indicated firefly luciferase reporter plasmid, 20 ng of Renilla reporter plasmid as a normalization control, and 25 nM of miR-34a or a negative control oligonucleotide. After 48 hours, a Dual Luciferase Reporter assay (Promega) was performed according to the manufacturer's instructions. Fluorescence intensities were measured with an Orion II luminometer (Berthold) in 96-well format and analyzed with the SIMPLICITY software package (DLR).
